# Small Ruminant Farming in Tribal Areas of Dera Ghazi Khan, Punjab, Pakistan

**DOI:** 10.3390/vetsci9060279

**Published:** 2022-06-07

**Authors:** Muhammad Ameen Jamal, Samiullah Khan, Yanhua Su, Chang Yang, Heng Zhao, Kaixiang Xu, Deling Jiao, Wenmin Cheng, Abdul Rauf, Mahboob Ali, Sohail Ahmad, Yubo Qing, Hong-Jiang Wei

**Affiliations:** 1 Yunnan Key Laboratory for Porcine Gene Editing and Xenotransplantation, Kunming 650201, China; drameen007@mail.kiz.ac.cn (M.A.J.); samiullahakbar4@gmail.com (S.K.); 2013003@ynau.edu.cn (Y.S.); theurgytheurgy@163.com (C.Y.); hengzhao2014@126.com (H.Z.); tsljmuch@163.com (K.X.); jiaodeling@163.com (D.J.); cheng_8097@163.com (W.C.); 2 Kunming Institute of Zoology, Chinese Academy of Sciences, Kunming 650201, China; 3 Faculty of Animal Science and Technology, Yunnan Agricultural University, Kunming 650201, China; 4 College of Veterinary Medicine, Yunnan Agricultural University, Kunming 650201, China; 5 Institute of Pharmaceutical Sciences, University of Veterinary and Animal Sciences, Lahore 54000, Pakistan; raufkaisrani@gmail.com; 6 National Veterinary Laboratory, Ministry of National Food Security and Research, Islamabad 45710, Pakistan; qaisbros@gmail.com; 7 Institute of Biotechnology and Genetic Engineering, The University of Agriculture, Peshawar 25120, Pakistan; sohail_abg@aup.edu.pk

**Keywords:** sheep farming, reproduction, health, Tribal Area Dera Ghazi Khan

## Abstract

Provincially Administered Tribal Areas (PATA) of Punjab-Pakistan are comprised of hilly mountains with small ruminants as a sole source of income. In this study, farming practices, productivity, health and the economic value of sheep were evaluated in PATA through a survey of farmers (*n* = 138) holding 11,558 heads of sheep. Out of a total population, 87% were non-descriptive flocks, and 9% and 4% were purebred flocks belonging to the Kajli and Thali populations, respectively. Sheep flocks were mainly (86%) reared under the traditional production system and had a delayed onset of puberty. There was low influence of season on the reproduction, and the majority of flocks (78%) were bred throughout the year. The lack of proper vaccination and poor management exposed the flocks to bacterial, viral and parasitic infections, which lead to high mortality in lambs (~22%) and adults (~32%). The share of sheep in farmers livelihood was 42%, and only 20% of producers’ living standard was improved with sheep farming, but the rise in rearing more sheep was quite low (20%). Although the livestock department arranged farmers’ training, the majority of farmers (83%) never participated in training and had no knowledge of modern technologies. Collectively, the traditional sheep production systems, poor management, lack of vaccination, marketing channels and farmers training hampered the sheep rearing and producers’ livelihood in the PATA of Punjab-Pakistan. However, developing model livestock farms, conducting farmer training, establishing a viable market for dairy products, and introducing subsidy policy interventions can improve the sheep farming in these areas.

## 1. Introduction

Agricultural development is an essential prerequisite for sustainable economic development and poverty alleviation [1]. About 75 percent of the world’s 1.2 billion poor (living on less than US $1 a day) inhabit rural areas and depend on the agriculture sector directly [2,3]. Agriculture plays a significant role in poverty reduction, especially in the rural areas which are deprived of resources [4]. It has been estimated that three-quarters of poorer households kept livestock as part of their livelihood portfolios [5]. Livestock mixed with crops are an important component of farming systems to strengthen and improve their operation and management for long-term productivity, profitability and sustainability in developing countries across the world [6].

Pakistan is also an agricultural country, with a 21% contribution to gross domestic product (GDP), while livestock is major sub-sector of agriculture with about an 11.9% contribution to national GDP [7]. Livestock has an important role to play in promoting socio-economic development, particularly in rural areas. Nearly eight million families are involved in livestock raising, from which they derive more than35% of their income [8]. Similar to other developing countries, the backyard rearing of small or large ruminants contributes to the livelihood of households in Pakistan. Like other parts of the country, particularly in Dera Ghazi Khan, a large number of households in rural areas are involved in livestock rearing [9]. Provincially Administrated Tribal Areas (PATA) of Punjab are located in the Dera Ghazi Khan District in the Sulaiman mountain ranges. It is rich in minerals like uranium, gypsum, marble, in addition to proven oil and gas reserves in the Dhodak and Baghalchurr area, but there is little economic activity [10]. In these areas the livestock, particularly small ruminants, are extensively reared as a potential source of income.

Sheep, with its multi-faceted utility for wool, meat, milk, skins and manure, form an important component of the rural economy, particularly in these mountainous areas. It provides a dependable source of income to the shepherds through the sale of wool and animals [11]. Furthermore, their high reproductive potential, short generation interval, ability to thrive on shrubs, bushes, and tree leaves, and high digestive efficiency for cellulose make them suitable as meat-producing livestock [12]. Unfortunately, PATA are severely lacking in health and education facilities and also have no efficient transportation system [13]. However, one study highlighted the significance of livestock in federally administered tribal areas of KPK province [14] but until now no report was available in the PATA of Dera Ghazi khan. Therefore, the objective of the current study was to evaluate the status of sheep farming practices, their productivity, health and economic value in Tribal areas and adjacent areas of Sulaiman Mountain.

## 2. Materials and Methods

### 2.1. Study Area and Design

**Figure 1:**
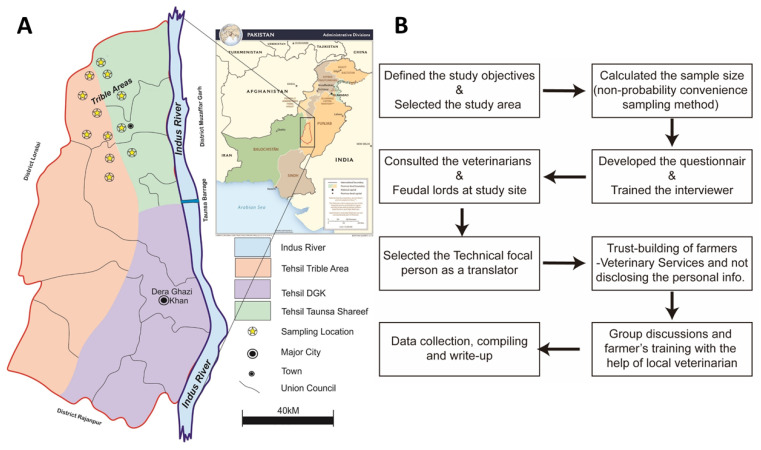
Sampling area (**A**) and survey building steps (**B**).

The current study was carried out in the Tehsil Taunsa Sharif and adjacent Tribal Area of district Dera Ghazi Khan. The district Dera Ghazi Khan covers an area of 8493 km^2^. The district is a long narrow strip of country, 317 km in length, sloping gradually from the hills which form its western boundary to the river Indus to the east. Below the hills the plain is high and arid, generally level, but sometimes rolling in sandy undulations and is intersected by 201 hill torrents. The sheep population of the district is 1.18 million. The Koh-e-Sulaiman (Sulaiman Mountains) constitutes a major part of this area, and is full of natural deposits like iron, gypsum, marble, limestone, cement, precious stones, and uranium, coal and petroleum and gas reserves. The small ruminant population in the Tribal area is 196,225 heads, while in Tehsil Taunsa sharif it is 234,337 heads (http://9211.punjab.gov.pk/census2017/index, accessed on 9 July 2021). There were three Mobile Veterinary Dispensaries in the Tribal area of tehsil Taunsa Sharif district Dera Ghazi Khan. A structured questionnaire was used to collect the information from the farmers (who were involved in livestock farming as their livelihood) living in the 12 union councils of Tehsil Taunsa Sharif and the adjacent Tribal areas shown in (Figure 1A). In order to capture the targeted information, the survey-building steps are shown in Figure 1B.

The focus of the current study was to evaluate the status of sheep farming practices, their productivity, health and economic value in Tribal areas and adjacent areas of the Sulaiman mountains. A pre-designed questionnaire was used for the collection of data. The questionnaire contained questions on the demographic aspects of the farmers and animal husbandry and management practices (Supplementary File S1). 

### 2.2. Sample Size and Sampling Unit

The sample size in the study area was determined on the basis of disease prevalence in District Dera Ghazi Khan [15], and was calculated as *n* = 138 farmers, according to [16] the non-probability convenience sampling method, because the list of farmers was not available and the study area was very large (11,922 km^2^). Each interviewed person was considered as the sampling unit. The sampling unit consisted of farmers that were rearing sheep as the main source of their livelihood. Each farmer was considered as a herd.

### 2.3. Statistical Analysis

To manage the data, a database was created in Microsoft Office Excel 2019, and all the data is presented as descriptive statistics (also available at Supplementary File S2).

## 3. Results

### 3.1. Flock Distribution and Production Systems

**Figure 2:**
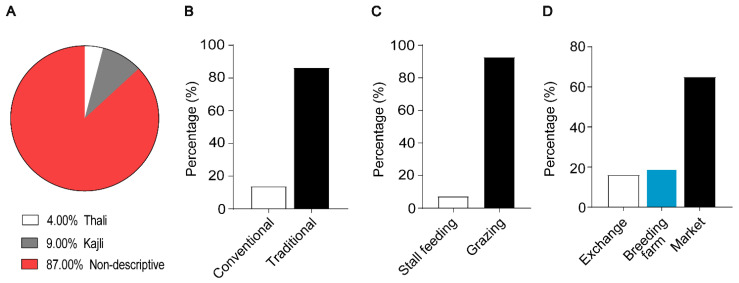
Sheep Flock distribution and production systems: (**A**) Flock distribution (**B**) Sheep production system (**C**) sheep feeding system. (**D**) Sheep source.

Out of total population, 87% were non-descriptive flocks; and nine percent and four percent were purebred flocks belonging to the Kajli and Thali population, respectively (Figure 2A). Eighty-six percent of sheep flocks were reared under traditional production systems (sheep raising in nomadic systems refers to tribesmen inhabiting the border areas of the country who inherited their animal resources and rearing practices over generations), while 14% were reared under conventional production systems (extensive or intensive farming systems where semi-open or bound housing systems were adopted for sheep rearing) (Figure 2B). Along with traditional farming systems, the 93% of flocks were reared on grazing, while there were also a few flocks (7%) reared on stall feeding (Figure 2C). Regarding the sheep source, it was observed that 65% of the total population was purchased from the nearby market (in the local language known as “Mandi”), while there were also practices of exchange between farmers and sometimes sheep were purchased from government breeding farms (Figure 2D).

### 3.2. Reproductive Status

**Figure 3:**
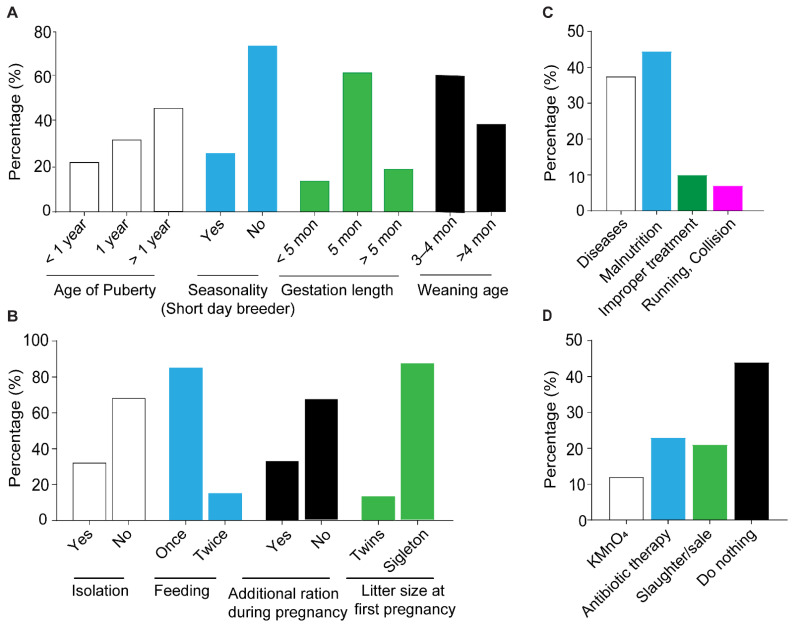
Reproductive performance: (**A**). Age of puberty, time of estrus expression, gestation period and weaning age (**B**) Feeding and housing management during pregnancy, and litter size at first lambing (**C**) Causes of abortion (**D**) Nursing after abortion.

We investigated the reproductive status of flocks using different parameters (Figure 3A), and it was observed that the onset of puberty was variable (10–15 months), with 22%, 32%; and 46% of flocks displaying estrus at the age of <one year (9–11 months), one year and >one year (13–15 months), respectively (Figure 3A). Although sheep are regarded as short day breeders (breeding at times of the year when the day length is shorter and the dark period is longer), the low influence of seasonality (influence of season on the estrus expression and breeding) was observed in these tribal areas, as 74% of flocks manifest heat and are bred throughout the year, while only 26% of flocks express heat during short days of the year (the winter season). Furthermore, the gestation period of 62% of ewes was five months (145~155 days), while 15% were <five months (130~145 days) and 23% were >five months (155~165 days). The weaning age was recorded as four months in 61% of flocks and >four months was also reported in 39% of flocks (Figure 3A). Regarding breeding and pregnancy management (Figure 3B), most farmers use adult breeding males (20–24 months old) for breeding, and during pregnancy 68% of ewes were not isolated from the flock and no special rations were offered during pregnancy. The birth of twins at first lambing was less, (13%) as the majority of ewes (87%) gave birth to a single lamb at first pregnancy (Figure 3B). Furthermore, mostly malnutrition (44.5%) and diseases (38.5%) were major causes of abortion, while improper treatment (like misuse of laxative or accidental steroid injections) was among the cause of 10% abortions (Figure 3C). There were also practices of caring for the aborted ewes by using antibiotics (23%) and antiseptics (12%). However, 44% of aborted flocks were deprived of any supportive therapy and 21% of aborted ewes were even culled (slaughter or sale out) from the flock with the fear of repeated abortion (Figure 3D).

### 3.3. Health Status

**Figure 4:**
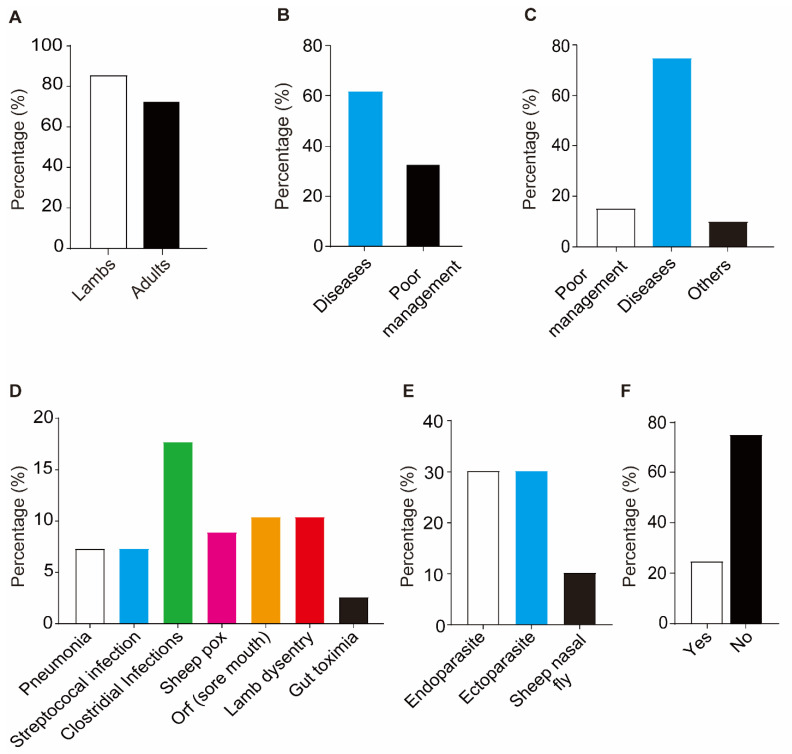
The health status. (**A**) Percentage of lambs and adults that survived. (**B**) Major causes of mortality in lambs. (**C**) Major causes of mortality in adults. (**D**) Prevalence of infectious diseases. (**E**) Prevalence of parasitic diseases. (**F**) Vaccination practices.

We then investigated the health status of the flock, and out of total births, the survival percentage in lambs was 78~88% (12~22% mortality), and in adults it was 68~82% (18~32%, Figure 4A). The main cause of death in lambs was disease (65%), while poor management such as the lack of colostrum feeding or insufficient colostrum feeding, or exposure to an adverse environment lead to 33% mortality in lambs (Figure 4B). Similarly, diseases were the main cause (75%) of death in adults along with poor management (15%) and other factors (5%) (Figure 4C). It was observed that the prevalence of clostridia diseases, of (sore mouth), pneumonia and streptococcal infections was 17.7%, 10.4%, 7.3% and 7.3%, respectively (Figure 4D). Among parasitic diseases, external and internal parasitic infestation was about 30%, with 6% nasal fly infestation (Figure 4E). Furthermore, it was also observed that there were no vaccination practices used, as about 25% of farmers responded towards vaccination (Figure 4E).

### 3.4. Farmers Income Sources and Status of Sheep Rearing

**Figure 5:**
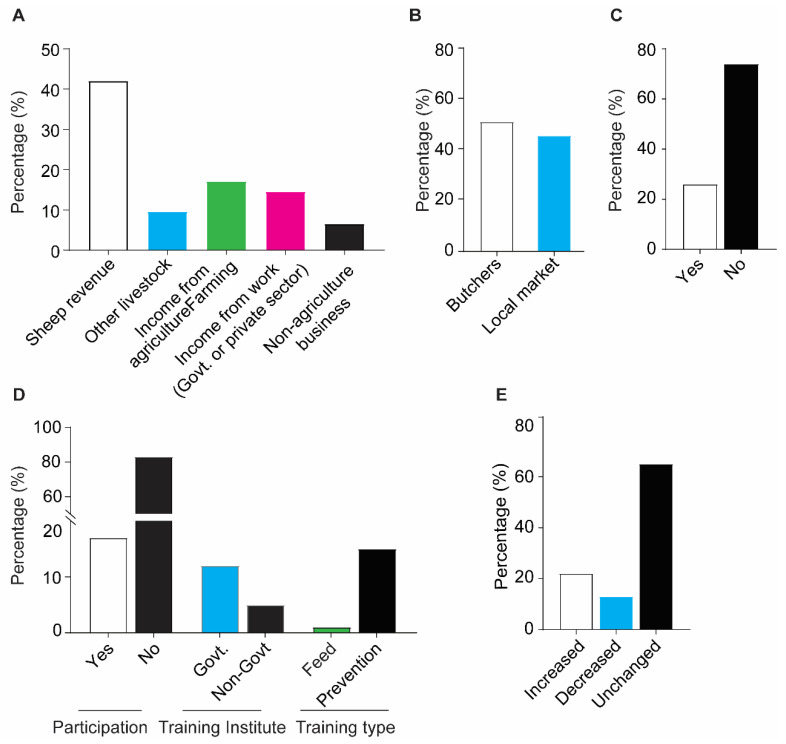
Sheep contribution to farmer’s livelihood and practices of rearing sheep. (**A**) Household income (**B**) Sheep sale channels (**C**) Improvement in livelihood due to sheep farming (**D**) Farmers training regarding livestock rearing (**E**) Practices of rearing more sheep.

In these hilly areas, most farmers exist on their farming income, and it was observed that about 42% of farmers derived their household income from sheep revenue, while the other major contribution was income from agricultural farming (17%), followed by 14.5% income from work (government or private sector) and about 9.5% from other livestock (including poultry and large animals) (Figure 5A). There was also a lack of proper marketing channels, as sheep were mostly sold to butchers (52.5%) and at nearby markets (47.5%, Figure 5B). Next, we evaluated the influence of sheep farming on the improvement in farmers livelihoods, and it was observed that only 26% of farmers were satisfied with sheep farming, while 74% found no improvement in their living standards from sheep rearing (Figure 5C). Furthermore, only 17% of farmers ever joined the training sessions that were mostly conducted by the government (12%), and this training was mostly about disease prevention (15%); however, one% farmers also joined the training regarding feeding (Figure 5D). Thus, these malpractices finally impacting the sheep rearing practices as only 22% farmers responded increment in rearing more sheep in local vicinity, while 65% of farmers responded no increment in rearing more sheep, however fewer (13%) responded decrease in rearing sheep in their local vicinity (Figure 5E).

## 4. Discussion

Provincially Administrated Tribal Areas of Punjab, located in Dera Ghazi Khan, are comprised of hilly mountains and are rural in nature. In the present subsistence farming system of the hills, farmers have little surplus agricultural products to sell and hence depend upon the livestock and their products as a source of income. However, because of their inherent ability to utilize mountain terrain that is unsuitable for crop farming, a high proportion of sheep and goats are found in the hills. Thus, small ruminants are reared by these landless people as a source of income as well as for security against difficult times [17,18]. Thus, in present study we evaluated the status of sheep farming systems, breeding and health practices and sheep contribution to livelihood in the tribal areas of Punjab, particularly in the district of Dera Ghazi Khan.

In the current survey, it was observed that most farmers reared non-descriptive breeds, while farmers inhabiting the nearby plain areas also had purebred flocks of Thali and Kajli breeds. A diversity of animals is reared in these areas, including cattle, buffalo, goats, sheep, donkey, horse, camel, mule and poultry [19]. Therefore, in the current survey, we observed that, along with sheep farming, the farmers also reared other livestock like large ruminants and poultry (data not shown). The traditional production system was mostly observed, with grazing of the flocks on natural grasses, herbs and shrubs. Also, there were no specific housing systems, as most flocks were housed on open ground fenced by trees and shrubs. Irrespective of grazing, no extra rations were provided to the sheep. Similar farming practices were already reported in the Baluchistan province of Pakistan [20], as well as in other countries [21,22] where most land is covered by mountains and hills. Reproductive efficiency is one of the main factors that determines the efficiency of production, especially in countries in which the sheep industry, and particularly meat production, is important [23]. In these tribal areas, the onset of puberty in the majority of the flock was delayed due to the poor nutritive value of the rations, as it has been reported that better nutrition induces the earlier onset of puberty [23,24,25]. Furthermore, no specific influence of seasonality on sheep reproduction was observed. This information was contradictory to the concrete finding that sheep are short (they express heat and can be bred in the season when daylight is short) [26,27]. However, no specific pattern of seasonality on sheep reproduction has been reported in another study conducted in the district of Dera Ghazi Khan in which 50% of flocks expressed heat throughout the year. Another study on the Lohi breed (Pakistan) also reported breeding throughout the years [28], and it could also be related to the presence of males, as it was observed that they were reared along with the females thus pheromones provokes estrus [29], hence ewes could be bred throughout the year rather than only in specific season. Furthermore, it has also been reported that a few breeds of sheep are also non-seasonal breeders [30]. Therefore, there is still a need to conduct survey across Pakistan to clearly differentiate the seasonal and non-seasonal breeds in the country. In addition, in these areas, abortions in ewes were mainly due to diseases, and several studies reported that infectious diseases like brucellosis [31] toxoplasmosis [32], and the peste des petitis ruminant [33] were prevalent in different areas of Pakistan that cause abortion in small ruminants. However, the prevalence of these infectious diseases and their contribution to abortions in these tribal areas is still unavailable. Thus, there is a need to conduct a precise survey regarding the identification and contribution of diseases causing abortion.

Next, we investigated the health status of the flock, and it was observed that infectious diseases were more prevalent (65%) than parasitic ones (Figure 4A). A lot of studies conducted in different areas of Pakistan highlighted the prevalence of infectious diseases including clostridia infections that causes enterotoxaemia, lamb dysentery leading to high mortality [34,35,36,37,38], foot and mouth disease (FMD) [39,40] and other streptococcal infections [41]. Similarly, internal and external parasite infestations up to 46% have also been reported in different areas of Pakistan [42,43,44]. These diseases causes severe mortality in the flocks, as we observed the survival percentage of lambs and adults to be 78~88% (12~22% mortality) and 68~-82% (18~32%), respectively, and the major causes of death were disease outbreaks (Figure 4C–E). Although the disease outbreaks were reported in various areas of the country, there were effective measures, vaccination schedules and deworming schemes that were effective in controlling disease outbreaks. In the current study, we did not observe strict vaccination practices and the proper care of diseased flocks. Although there were mobile veterinary dispensary serves available, most farmers were more interested in fattening their sheep rather than preventing disease. Hence, they inquired about anthelmintic rather than vaccines, and there were also myths about vaccination stress to animals and even about the efficacy of vaccines. It is worthwhile to mention that vaccination failure could be due to either ignorance in cold chain maintenance or to the lack of a proper schedule for vaccination, as many farmers start vaccinating during disease prevalence, which ultimately leads to vaccination failure. Therefore, this kind of negligence, as well as improper management, results in the serious mortality rates in the flock and thus huge losses to farmers.

Livestock is the key component of the rural economy, as it satisfies the household needs for milk, yoghurt, butter and whey [45]. We next evaluated the livelihood of farmers in these hilly areas and observed that they were involved in rearing other livestock along with sheep. In order to fulfill their needs, they were also involved in many other business like agricultural farming, official jobs and non-agricultural businesses. Collectively, the sheep’s share of the household income of all peoples inhabiting these hilly mountains was about 42%. A similar source of income was already reported in the peoples of the Federally Administered Tribal Areas (FATA) of Pakistan, but in those areas the share of total livestock was about 19% [14]. There is a growing realization that sometimes unique information can be gained by using structured questionnaires to solicit the experiences of those involved, which is of particular value for researchers from many disciplines [46,47]. Many investigators reported only the experiences and opinions of those farmers and communities having little or no scientific background [48,49,50]. In our present survey, we first time-collected the basic information on sheep farming from the farmers inhabiting the tribal areas of Punjab Province and called it a descriptive study, as it was questionnaire-based. However, there is a need to conduct a more detailed analytical study in the future with a particular focus on the financial contributions of livestock to household income; sources of income; their annual expenses (PKR: Pakistani Currency); income generated from the different sources (sheep, other livestock, or other business); utilization of livestock resources at home (milk, yogurt, butter etc.). It was obvious to observe that most of the farmers were not satisfied with sheep farming, and thus the trend of rearing more sheep and the improvement in their living standards remained unchanged. Such unsatisfactory and unprofitable sheep farming could be due to their traditional production systems, the improper care and management of flocks, the lack of effective preventive measures against diseases and malnutrition, and the lack of scientific knowledge about sheep farming and husbandry.

However, in order to improve the livestock production in such areas, the following measures are suggested:

1. Efforts must be made to establish the small viable purebred sheep units in the selected areas, and purebred breeding rams must be made available to propagate the purebred flocks to produce more meat and wool. Proper sale channels should be set up to facilitate the sale of farmer’s products to large cities. The establishment of model sheep farms in the tribal areas and the provision of farm training to young sheep farmers may help with the increased adoption of modern sheep husbandry practices.
2. Educational activities such as the organization of mass media programs like deworming, vaccination and sheep health camps by the extension agencies can efficiently disseminate scientific knowledge to the sheep farmers. In addition, the farmers should be properly protected against vaccination myths and cold chains must be maintained to retain the efficiency of vaccines.
3. In order to cope with malnutrition, the government waste Banjar lands in the villages should be distributed to the landless sheep farmers’ community for the encouraging of fodder cultivation.

In sum, the traditional production system, malnutrition and poor management in PATA, Punjab, Pakistan led to late puberty and weaning in the flocks. Furthermore, the lack of vaccination and poor management exposed the flocks to infectious diseases and thus caused mortality in lambs and adults. The lack of scientific knowledge and farmers training made the sheep farming unprofitable in these hilly mountains.

## 5. Limitations of the Study

The following challenges/limitations were observed while conducting the survey:

1. Communication barriers: There were communication barriers between the farmers inhabiting the mountain areas and interviewers because the questionnaire was written in English and Urdu (the official language of Pakistan), but most of the farmers were illiterate. Although the questionnaire was explained to the farmers with the help of veterinarian in the local language, it is possible that there were errors in translation
2. Some respondents were skeptical about revealing accurate figures relating to the number of their livestock; this was avoided by emphasizing the confidentiality of information during the interviews.
3. Many of the questions in the questionnaire were based on the recall ability of the respondents, who may not have given very accurate information due to memory lapses, considering that most of them even do not even have a basic education.
4. The lack of producer records and social mistrust with regard to providing information was also observed during the survey.
5. This study focused on descriptive research, so there is a need for more analytical measurements

## Data Availability

All the data used in the manuscript is available in Supplementary File S2.

## Supplementary Materials

The following supporting information can be downloaded at: https://www.mdpi.com/article/10.3390/vetsci9060279/s1, File S1: Questionnaire used for data collection in this study; File S2: Data used in this study.
