# Supplementary material for: Small Ruminant Farming in Tribal Areas of Dera Ghazi Khan, Punjab, Pakistan
Source: Vet Sci. 2022 Jun 7;9(6):279. doi: 10.3390/vetsci9060279 (PMC9229448; doi:10.3390/vetsci9060279)
Supplement: Supplementary file 1 [file vetsci-09-00279-s001.zip › vetsci-1682049-supplementary.pdf]

veterinary sciences

MDPI

Supplementary File S1: Questionnaire used for data collection in this study.

Survey Number:__________

Current status of sheep farming in Tribal areas of Pakistan

Questionnaire

Dear sheep farmers:

Hello! We are researchers from Yunnan Agricultural University, Kunming, Yunnan Province, China and had a collaborative project with Agriculture University Peshawar. We were interested in conducting a survey about sheep farming in Tribal area of Pakistan. The information will be only used for current research project without any adverse effects on object. Therefore, it is requested to provide accurate information. Thanks a lot for your support and cooperation!

1) Basic status of sheep rearing

1. Your area in Pakistan ____________________.
2. Breeds of sheep in your area_____________________________________
3. Type of farming
   a) Conventional/ Commercial Farming
   b) Traditional Farming
4. Feeding system_________
   a) Grazing
   b) Stall Feeding
5. Housing system
   a) Open
   b) Semi-closed
   c) Closed
6. Sheep Source__________
   a) Exchange
   b) Breeding Farm

c) Purchased from market

7. In addition to rearing sheep, the other livestock you also raise
    a) Cattle and Buffalo
    b) Poultry
    c) Horse and donkey
    d) None
8. Flock composition ……………………………

2) Reproductive status

9. In which season sheep normally display heat signs………………………
10. Average breeding age of sheep (months)_________________,.
11. Number of lambs at first pregnancy

    A. single lamb **B**. Twins **C.** Three lamb and more

12. The average gestation period is ewes________,

    A. <5mon B. 5mon C >5mon

13. Pregnancy management (multiple choice)_____________

    A. Isolation B. Clustering

14. Feeding management during pregnancy

    Feeding times (per day) **A**) One time B) Two times C) none

15. Additional ration provided

    A) Yes B) No

16. The number of aborted ewes____________and cause of abortion

    A).Malnutrition

    B). Due to run, collision, crowded, shock caused by the abortion

    C). Feeding mildew, deterioration, frozen feed

    D). Disease

    F). improper treatment (such as: misuse of laxatives to promote a large number of severe contraction of smooth muscle

    G) Other reasons

17. Nursing sheep after abortion (more)______________

a) Washing the uterus potassium permanganate

b) Injection of Antibiotic

c) Slaughter or sale

d) Do nothing

3) Health Status

18. Disease infection

		Disease I	Disease II	Disease III	Disease IV
Infectious disease	Name of disease or condition				
	The number of infected sheep				
	Expenditure (PKR)				
Parasitic disease	Name of disease or condition				
	The number of infected sheep				
	Expenditure (PKR)				
Common disease	Name of disease or condition				
	The number of infected sheep				
	Expenditure (PKR)				

NOTE: As is known in the name of disease, the disease may be filled directly into a respective code; as the name of disease is unknown, can fill performance disorders, such as vomiting, shaking his head like.

▲infectious disease:

a. Sheep Mycoplasma pneumonia

b. Streptococcal disease

c. Clostridial disease

d. .Sheep pox

e. Orf

f. .Lamb dysentery

g. .Gut toxemia

h. other

▲parasitic disease:①.Gastrointestinal worm disease ②.tapeworm ③.Scabies ④.Ticks ⑤.Sheep nasal fly ⑥.protozoan ⑦.other

19. The most harmful disease _

a) Infectious disease

b) Parasitic

c) Both

20. Susceptible flocks__________

a) . Lamb

b) Breeding ewes

c) Adult sheep

21. Main causes of death in adult sheep_________

a) Poor Management and improper care

b) Diseases,

c) other

22. The main reason of death of the Lamb__________

a) Innate

b) Poor management and improper care

c) C. Diseases,

d) do not know

23. The survival of lambs____%,

24. Survival of adults_________%,

25. Is there a free immunization programs__________

    a) No

    b) Yes;

    c) the project name______________

26. Household income in turn derived from more to less________>________>________>________>_______.

    a) Sheep Revenue

    b) Other livestock income

    c) Agricultural farming income

    d) Income from work (Government or private servant

    e) Income from business other than agricultural farming or job

    f) Others

27. Sheep income ratio of total household income is_____________%.

28. The main sales channels__________

    a) Sold to butchers

    b) Self-slaughter from the sale

    c) Meat processing or marketing cooperatives

    d) Near Market

29. Usually, at which time the slaughtering of sheep is performed in 2018

    a) At Special Festival like EID UL AZHA

    b) At marriage

    c) Others

30. The trend of sheep farming in your vicinity is __________________

    a) Increased

    b) Decreased

    c) Unchanged

d) I do not know

31. Have ever participated sheep farming related technical training__________

a) Yes

b) No

32. Training types (multiple choice)__________

a) Feeding Technology

b) Prevention

c) Management

d) Marketing information.

33. Breeding and training institutions__________

a) Government departments

b) Cooperative Organization

c) Enterprise

34. Feeding way to get technical information________

a) Other farmers
b) .Promotion agencies
c) CCTV
d) Website
e) books
f) Technical staff
g) other

35. Being neighbor and best friend country What's expectation from China _________

a) Multiplication

b) Nutrition

c) Disease prevention and treatment

d) Production management

e) Slaughter and sale.

36. Have you heard of the new breeding techniques such as superovulation, in vitro fertilization and embryo transfer technology?

    a) Yes

    b) No

37. Have you used the new reproductive technologies such as superovulation, in vitro fertilization and embryo transfer technology?

    a) Yes

    b) No

Supplementary File S2: Data used in this study

**Fig 2:**	
Sheep Breeds	%
Thali	4
Kajli	9
Non-descriptive	87

**Fig 2: Flock distribution and husbandry**		
Parameter	Category	% of respondent
Type of farmers	Conventional	14
	Traditional	86
Feeding system	Grazing	93
	Stall feeding	7
Housing system	Open	89
	Closed	11
Usually, at which time the slaughtering of sheep is performed	EID	25
	Marriage	12
	Other festivals	6
	EID & marriage	57
Sheep Source	Exchange	16
	Breeding Farm	19
	Purchased from Market	65

**Fig3: Reproduction**		
Parameter	Category	Response of respondent %
Breeding Age	< 1 year	22
	1 year	32
	> 1 year	46
Breeding time (months)	Winter Season	26
	All the year	74
Gestation length	<5 months	15
	5 months	62
	>5 months	23
Weaning age	3-4 months	61
	>4months	39
Pregnancy management	Isolation	32
	Clustering	68
Feeding management during pregnancy	One time	85
	2 time	15
Additional ration provided	Yes	33
	No	67
The Major cause of abortion of ewes	Malnutrition	23
	Diseases	17
	Improper treatment	10
	running, collision	7
	Malnutrition & Diseases	43
Nursing sheep after abortion (more)______________	Washing with KMnO4	12
	Treatment with Antibiotic	23
	Slaughter/Sale	21
	Do nothing	44
Number of lambs at first pregnancy	Single	87
	Twins	13

**Fig4. Disease**		
Parameter	Category	Response of respondant %
Infectious disease	Sheep Mycoplasma pneumoniae	7.3
	Streptococcal disease	7.3
	Clostridial disease	17.7
	Sheep pox	8.9
	Orf	10.4
	Lamb dysentery	10.4
	Gut toxemia	2.6
Parasitic disease	Gastrointestinal worm disease	15.1
	Ticks & Mites	15.1
	Sheep nasal fly	5.1
% of lambs remain alive	75%	9
	80%	14
	90%	51
	95%	26
% of adults remain alive	70%	25
	80%	46
	90%	29
The most harmful disease	Infectious disease	64
	Parasitic	36
immunization	Yes	25
	No	75
The main reason of death of the Lamb	Improper Management and Care	33
	Disease	65

The main reason of death of the Lamb	Improper Management and Care	15
	Disease	75
	Others	10

**Fig 5. Farmers livelihood**		
Parameter	Category	Response of respondant %
The trend of sheep farming in your vicinity is	Increasing	22
	Decreasing	13
	Unchanged	65
Whether sheep farming can improve the quality of family life	Yes	26
	No	74
The main sales channels	Sold to butchers	17
	Self-slaughter from the sale	13
	Near Market	70
Household income	Sheep Revenue	5
	other livestock	19
	Sheep, & Farming income	34
	Sheep & Income from work	29
	Sheep & Non-agricultural business income	13
	Sheep income ratio of total household income is	31
Whether sheep farming can improve the quality of family life	Yes	26
	No	74

Have ever participated sheep farming related technical training	Yes	17
	No	83
Training Type	Feeding Technology	1
	Prevention	15
Training Institution	Government	12
	Non Government	5
